# Boring sponges, an increasing threat for coral reefs affected by bleaching events

**DOI:** 10.1002/ece3.452

**Published:** 2013-03-04

**Authors:** José L Carballo, Eric Bautista, Héctor Nava, José A Cruz-Barraza, Jesus A Chávez

**Affiliations:** 1 Instituto de Ciencias del Mar y Limnología (Unidad Académica Mazatlán), Universidad Nacional Autónoma de MéxicoAvenida Joel Montes Camarena s/n, PoBox 811, Mazatlán, 82040, Sinaloa, México; 2 Posgrado en Ciencias del Mar y Limnología, Universidad Nacional AutÓnoma de México, Avenida Joel Montes Camarena s/n, PoBox 811 Mazatlán, 82040, Sinaloa, México

**Keywords:** Bioerosion, bleaching, boring sponges, climate change, coral reefs, Eastern Pacific Ocean

## Abstract

Coral bleaching is a stress response of corals induced by a variety of factors, but these events have become more frequent and intense in response to recent climate-change-related temperature anomalies. We tested the hypothesis that coral reefs affected by bleaching events are currently heavily infested by boring sponges, which are playing a significant role in the destruction of their physical structure. Seventeen reefs that cover the entire distributional range of corals along the Mexican Pacific coast were studied between 2005/2006, and later between 2009/2010. Most of these coral reefs were previously impacted by bleaching events, which resulted in coral mortalities. Sponge abundance and species richness was used as an indicator of bioerosion, and coral cover was used to describe the present condition of coral reefs. Coral reefs are currently highly invaded (46% of the samples examined) by a very high diversity of boring sponges (20 species); being the coral reef framework the substrate most invaded (56%) followed by the rubbles (45%), and the living colonies (36%). The results also indicated that boring sponges are promoting the dislodgment of live colonies and large fragments from the framework. In summary, the eastern coral reefs affected by bleaching phenomena, mainly provoked by El Niño, present a high diversity and abundance of boring sponges, which are weakening the union of the colony with the reef framework and promoting their dislodgment. These phenomena will probably become even more intense and severe, as temperatures are projected to continue to rise under the scenarios for future climate change, which could place many eastern coral reefs beyond their survival threshold.

## Introduction

Climate-driven coral bleaching has emerged as one of the greatest threats to coral reef ecosystems (Hughes et al. 2003; Manzello et al. 2008). This phenomenon has been explained by diverse natural factors, but widespread bleaching events have been strongly correlated (in time and intensity) with rapid increases in sea surface temperatures (SSTs) to levels above the coral's thermal tolerance threshold during the El Niño events (Glynn 2000; Goreau et al. 2000), which exert serious physiological stress on this symbiotic association resulting in disastrous, massive coral mortalities (Glynn et al. 2001).

Eastern Pacific coral reefs extend from approximately 24°N, near the tip of Baja California (Mexico), to 2°S, including the mainland coast of Ecuador and the southern Galapagos Islands (Glynn and Ault 2000). These are very fragile reefs mainly constructed by interlocking branches of species of the genus *Pocillopora*, which usually develop in bays or along shores protected from strong swells. A brief historical revision shows that main coral bleaching in the Eastern Pacific coral reefs has been related to the El Niño Southern Oscillation (ENSO) (Glynn et al. 2001; Pari et al. 2002), a phenomenon dating back to the 1982/83 El Niño event, the most severe on record which devastated coral reefs worldwide, and caused the loss of 50–99% of coral reefs in this area (Glynn 1990; Guzmán and Cortés 1992; 93, Carriquiry et al. 2001). Another of the most extreme and most geographically extensive events in recent history was 1997/98 El Niño, which caused severe loss in coral reefs from the Indian Ocean, Caribbean, and particularly from Eastern tropical Pacific (Rohan 2000; Glynn et al. 2001; Riegl 2001; Reyes-Bonilla et al. 2002). This could mean the unavoidable degradation of these coral reefs and even their total destruction, since once dead, physical forces (mainly severe storms) and bioerosion destroy the complex three- dimensional coral reef framework into unconsolidated rubble (Sheppard et al. 2002). Some well-documented examples come from the Galápagos Islands, where 1000- to- 5000- year- old reef framework accumulations affected by the 1982/83 El Niño were reduced to rubble and sand by bioerosion of different organisms, such as fishes, echinoids, lithophagine bivalves, and boring sponges (Glynn 1990, 2000; Guzmán and Cortés 1993). However, one of the most outstanding components of the boring community taking part in the complex balance between accretion and erosion in coral reefs are the sponges (Goreau and Hartman 1963; Risk and Sammarco 1982; Holmes 2000). They can remove up to 22 kg CaCO_3_/m^2^/year of calcareous material from the coral reef framework, generating up to 40% of the sediment deposited on coral reef ecosystems (Neumann 1966; Rützler 1975), and accelerating the dissolution of carbonate in the water column (Zundelevich et al. 2007; Nava and Carballo 2008). They also can weaken the coral reef by causing dislodgment of coral colonies during hurricanes (Stearn and Scoffin 1977; MacDonald and Perry 2003; Carballo et al. 2008). Furthermore, the weakened substratum may also accelerate the activity of secondary borers (Hutchings 2008).

In addition, under stressful conditions, unlike corals, boring sponges appear to continue to function normally or can even occasionally become epidemic (Rose and Risk 1985; Vicente 1990), as some of these species harbor symbionts that display a more stable photochemical efficiency during and after thermal stress than corals (Schönberg et al. 2008). Moreover, temperature increase may promote boring sponge growth, thus aggravating the destruction of the reefs (Rützler 2002; Márquez et al. 2006), and eutrophication and high organic loads, that also can be detrimental for coral reefs, act positively for some sponges (Holmes et al. 2000). For example, *Cliona delitrix* increases their biomass in response to the increase in bacterial biomass due to the discharge of untreated fecal sewage (Rose and Risk 1985), and *Cliona lampa* (Ward-Paige et al. 2005) and *Cliona inconstans* (Cuet et al. 1988) do it due to the increase in nutrient input.

Thus, the ecology of the reef corals, and the modeling of the reef edge are all strongly influenced by boring sponges (Goreau and Hartman 1963; Carballo et al. 2008).

In the Eastern Pacific coast, 46% of the total distribution of coral reefs spreads out along the Mexican Pacific coast, and some of the most severely damaged coral reefs by El Niño events are in this region (Carriquiry et al. 2001; López-Perez and Hernández-Ballesteros 2004). It is well-known that boring sponges from the Mexican Pacific coast invade different calcareous substrata (Carballo et al. 2004, 2007; Bautista-Guerrero et al. 2006; Cruz-Barraza et al. 2011), which suggests that they could play an important ecological function on these coral reefs, and this also highlights the need to know their role in the current decline of these ecosystems. Given that coral-bleaching events have become more frequent and intense in response to recent climate-change-related temperature anomalies, and it will probably become even more intense and severe, as temperatures are projected to continue to rise under the scenarios for future climate change (IPCC 2007), we think that it is important to know the status of boring sponge assemblages in coral reefs, especially in coral reefs strongly impacted by past bleaching and El Niño events. We also aimed to study if coral reef degradation has been determinant for the increase in boring sponge abundance at a regional scale, in the last 6 years.

## Material and Methods

### Study area

**Figure 1:**
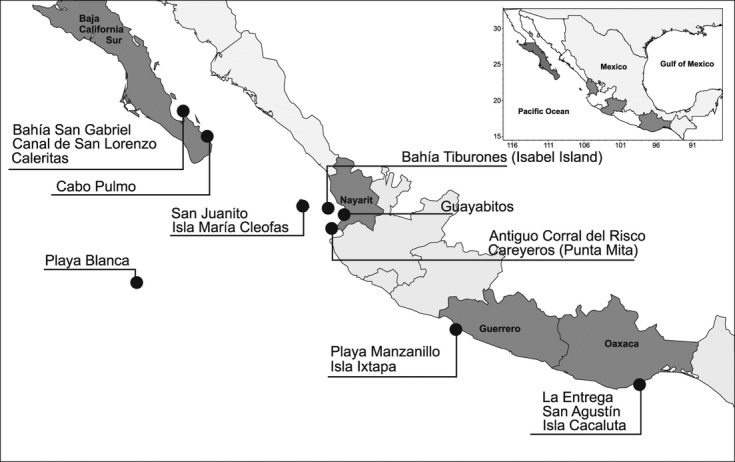
Sampling locations along the Pacific coast of Mexico

This study was carried out on 17 coral reefs and coral communities (areas with scattered colonies growing directly on bedrock) distributed along the Mexican Pacific coast (Fig. 1). The term framework is used following the definition of Fagerstrom (1987) as “the mass of large, colonial or gregarious, inter-grown skeletal organisms in general growth position.” Reef framework development is only considered to take place if at least one generation of corals has grown on the framework or rubble of at least one previous generation of corals.

The most important coral reefs in terms of their state of conservation and extension are in Oaxaca and Guerrero (southern region of Mexico) (Nava and Ramírez-Herrera 2011). We also have chosen coral reefs which have suffered strong impacts caused by bleaching events, such as Cabo Pulmo and Punta Mita (north Mexican Pacific) (Carriquiry et al. 2001; Reyes-Bonilla 2001), and some of them are currently completely decimated, such as Careyeros (central Mexican Pacific), where only some remains of the original framework exist.

#### Oaxaca reef area (15°40′–15°46′N, 96°03′–96°13′W)

The Bahías de Huatulco harbors some of the most developed coral reef structures in the Eastern Pacific coast, with 10 species of scleractinian corals (Glynn and Leyte-Morales 1997), mainly *Pocillopora damicornis*, *P. capitata,* and *P. verrucosa*. The coral reefs we studied are located at the bay of La Entrega, Cacaluta Island and San Agustin, where coverage of living corals occupies approximately 60% of the available substrata (Table 1). Cold water currents in winter produced by northern winds in 1998 have been related to episodes of high coral mortality in the area (Reyes-Bonilla et al. 2002), but the seasonal upwelling in the Gulf of Tehuantepec protected these coral reefs from the 1997/98 ENSO event (Glynn et al. 1998; Reyes-Bonilla et al. 2002). The ENSO event of 2002/03 reduced the coverage of living corals in the shallow part of La Entrega reef (McPhaden 2004). In this area, San Agustín, La Entrega, and Cacaluta reefs were sampled.

**Table 1:** Variation in coral coverage of some Mexican coral reefs, and sponge abundance and diversity

	Coral coverage								Sponge abundance		
				
State	Reef	1991	1998	2001	2003	2005	2010	Framework extension (ha)	2005–2006	2009–2010	Species
Oaxaca	La Entrega		76^(5)^		41^(6)^			7.5^(4)^	50	55	5
	Isla Cacaluta		90^(5)^		61^(6)^			1.7^(4)^	41	*	9
	San Agustín		73^(5)^		64^(5)^			2.5^(4)^	31	47	7
Manzanillo	Isla Ixtapa						24^(13)^	6.9^(13)^	*	14	6
	Playa Manzanillo						36^(13)^	0.5^(13)^	*	15	6
	Playa Blanca					13^(13)^	12^(13)^		38	41	13
Nayarit	Careyeros	44^(2)^	22^(3)^	3^(3)^	1^(13)^	0^(13)^	0^(13)^	83^(2)^	77	**	12
	Antiguo Corral del Risco	38^(2)^	33^(3)^	3^(3)^		7.5^(13)^	6.5^(13)^	0.001^(11)^	41	52	12
	Guayabitos					0^(13)^	0^(13)^		55	57	7
	Bahía Tiburones				8.6^(7)^	6.7^(13)^	6.5^(13)^	0.2^(13)^	47	55	12
	María Cleofas					49^(13)^			*	60	13
	San Juanito		40^(8)^	42^(8)^					*	63	8
	María Madre Sur		45^(8)^	40^(8)^					*	61	10
Baja California	San Lorenzo		40^(12)^			47^(13)^		0.3^(13)^	32	52	12
	San Gabriel					99^(13)^	80^(13)^		22	28	10
	Cabo Pulmo	30^(11)^	29^(3)^	30^(9)^	12^(1)^		18^(13)^	150^(9)^	56	58	11
	Caleritas						74^(13)^		37	32	8

Not much information about reef dimensions and coral coverage exist prior the 1997/98 bleaching event. The last three columns show the mean boring sponge abundance (%) at the beginning of the study (2005–2006) and at the end (2009–2010), and the number of boring species in each reef.

* Not sampled.

** Sampled, but no rest of reef or matrix framework was found.

1. Alvarez-Filip and Reyes-Bonilla 2006; 2. Carriquiry and Reyes-Bonilla 1997; 3. Carriquiry et al. 2001; 4. Leyte-Morales, 2001; 5. Lirman et al. 2001 6. López-Pérez et al., 2007; 7. Medina-Rosas and Cupul-Magaña 2004; 8. Pérez-Vivar et al. 2006; 9. Reyes-Bonilla and Calderon-Aguilera 1999; 10. Reyes-Bonilla and Calderon-Aguilera 1999; 11. Reyes-Bonilla 1993a; 12. Reyes-Bonilla 1993b; 13. This study.

#### Manzanillo & Guerrero reef area (17°37′–17°40′N, 101°31′–101°38′W)

The coastal area of Zihuatanejo (Guerrero) harbors important coral reefs with at least 10 species of scleractinian corals, mainly belonging to the genus *Pocillopora* (Reyes Bonilla et al. 2005). The fringing coral reefs of Playa Manzanillo and Ixtapa Island were preferred for this study due to their high coral coverage of living corals, and extension. Although no evident impacts of past ENSO events have been found, major sources of coral reef degradation at this area are high sedimentation derived from land erosion and mechanical damage, as a result of recreational activities such as scuba diving (Nava and Ramírez-Herrera 2011).

#### Nayarit reef area (20°46′–21°52′N, 105°16′–105°54′W)

They were some of the most diverse coral communities on the Mexican Pacific coast until the ENSO event of 1997/98, which caused massive mortality of corals in Bahia Banderas (up to 96%) (Carriquiry et al. 2001). At these sites, there is a record of 10 coral species also dominated by the genus *Pocillopora*. The Careyeros fringing reef extended up to 1.5 km along the shore and occupied approximately 83 Ha before the 1997/98 ENSO (Carriquiry and Reyes-Bonilla 1997). Today, it has practically dead coral substrata. The coral reefs of San Juanito and María Cleofas Island represent a very diverse area, with at least 20 coral species and a living coral coverage up to 40% (Pérez-Vivar et al. 2006). At these Islands, this ENSO event was related to a strong bleaching episode in corals from the sea surface to 20 m depth. Nonetheless, coral mortality was not recorded at this time (Reyes-Bonilla et al. 2002). Other reefs studied were the Antiguo Corral del Risco in Punta Mita, Guayabitos, Bahía Tiburones (Isabel Island), and a small patch south of the island Maria Madre. Some of these reef are have also endured bleaching episodes during El Niño-neutral years associate with upwelling and cold water (Cupul-Magaña and Calderón-Aguilera 2008; Rodríguez–Troncoso et al., 2010).

#### Baja California Sur reef area (24°25′–23°23′N, 110°21′–109°24′W)

This area harbors important coral reef formations in good conservation in San Lorenzo and San Gabriel Island, and Caleritas, where corals of the genera *Pocillopora* are the main reef builders and cover up to 99% of the available substrata. These three reefs, together with Cabo pulmo reef, were sampled during this study. Until the decade of the 90s, the coral reef of Cabo Pulmo reached more than 150 Ha, and it was known as the most important in the Mexican Pacific coast. At this site, coral diversity reached 10 species and the living coral coverage was 30–70%, mainly dominated by pocilloporid corals (Reyes-Bonilla 1993a). This coral reef lost a significant part of its framework after the El Niño event of 1997/98 (Reyes-Bonilla 2001). The reef of this area, particularly those closed to the Paz, have also suffered recurrent bleaching events associate with cold water (Hernández et al. 2010; Paz-García et al. 2012).

#### Socorro reef area (Archipelago of Revillagigedo) (19°34′–18°45′N, 111°3′–111°00′W)

It is a group of four volcanic islands; Socorro is the main island with at least 15 species of scleractinian corals (Ochoa-López et al. 1998). *Porites lobata* and pocilloporid corals are the major framework builders in the island. The isolation of the location has permitted it to avoid anthropogenic impacts, and the ENSO event of 1997/98 caused loss of coloration, but not coral mortality. However, more than true coral reefs, there are abundant coral patches (Ketchum and Reyes-Bonilla 1997), and there only exists a small reef in Playa Blanca, with a framework that is 3 m high.

### Water temperature

The relation of corals to water temperature and bleaching led us to start a monitoring program in one of the reefs studied (Bahia Tiburones, Isabel Island), which is representative of the central Mexican Pacific coral reef area. It was only possible to maintain this program in this reef because we were able to revisit it periodically (due to logistical and economical reasons). Thus, at the start of this study, we placed two HOBO Water Temperature devices at 6 m depth in the center of the reef, programmed to record water temperature every 6 h, as temperature loggers positioned within the marine communities are more representative of the water temperatures affecting the coral reef than satellite generated data (Davis et al. 2007). Monthly SST data (from 2005 to 2012) were extracted from the global dataset for each area, derived from the analysis of NOAA environmental satellite AVHRR thermal imagery. Temperature data corresponded to geographic coordinate of each area (see Study area section).

### Acquisition of field data

We have compiled information from cover surveys of Mexican reefs from a range of sources. In addition, most of the reefs were surveyed from 2005 to 2010. These data are summarized in Table 1. To describe the present condition, coral reefs was used, coral cover, a general indicator in coral reef assessment programs and critical for establishing baseline data (Rogers et al. 1994). For that, we used from 6 to 18 video-transects 25 m long according to the area occupied by the reef, which were haphazardly located at the top of each coral reef (between 2 and 5 m depth). Later, a high-resolution digital video-camera protected by a housing case was used to film along each one. A diver swam at a constant speed holding the camera downwards at approximately 1 m away from the subject (Moore et al. 2003).

### Analysis of images

The movies were later transferred to the computer, using the Windows Movie Maker software to produce a frame with approximately 800 Kb resolution (photography) at approximately every 5-s of filming. Thus, a subset of approximately 40–50 random nonoverlapping images per transect were extracted, which represent approximately one image each 0.5 m interval. This method resulted in a coverage area of 10–12 m² per transect, which is enough to document changes in reef cover (Haralson 2006).

Each image was analyzed using CPCe (Coral Point Count with Excel extensions) software developed by the National Coral Reef Institute (FL), using 100 random dots. This program was specially developed to meet the needs of the video-transect method (Kohler and Gill 2006).

### Quantification of the frequency of invasion by boring sponges

**Figure 2:**
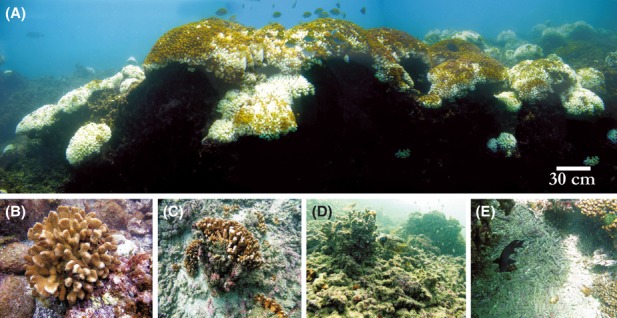
(A) General view of the Bahía Tiburones reef (Isabel Island) 2 months after the onset of a massive bleaching (November 2009). Corals partially covered by seaweeds. Classification of the coral substrata used in the study. (B) Attached live coral (ALC) (C) Detached live coral (DLC), (D) Coral reef framework (CRF). (E) Coral rubbles (CR).

Six transects 50 m long were randomly placed on each reef, and along each one, a coral rubble (CR), a small fragment of the coral reef framework (CRF), and a complete branch of a live coral colony (LCC) (from the tip to the stem) from the colony closest to the transect, were collected at random, approximately every 2 m, yielding 75 pieces per transect (25 pieces of each category) (Carballo et al. 2008*;* Nava and Carballo 2008, 2013) (Fig. 2). We also estimated the frequency of invasion in live colonies recently detached from the matrix (a colony that is partially alive and detached from the substrate). For that, on the periphery of each reef, we tried to look for at least 25 recently detached colonies (replicated in three different areas of the reef), and small fragments of their stems were collected. As we did not always find colonies recently broken away from the matrix, this information is only available in some reefs. All the fragments collected were broken into small pieces at the laboratory looking for boring sponge presence. When a sponge was found, a small portion of its tissue was digested with sodium hypochlorite and its spicules were examined under an optical microscope (Carballo et al. 2004). The occurrence/nonoccurrence of boring sponges in each piece was expressed as percentage of invasion (%). Thus, the abundance of boring sponges per coral reef was obtained by averaging the percentage of invasion of all the transects (mean ± SD) considering the 75 pieces per transect as 100% of invasion. The abundance of boring sponges by substrata was obtained considering 25 pieces of each category as 100% and later averaging the total of transects per reef (mean ± sd). Species richness of boring sponges was also recorded per coral reef and substrata type.

### Statistics analyses

Differences in coral invasion by boring sponges over time were analyzed using a one-way analysis of variance (ANOVA) after verifying normality (Kolmogorov–Smirnov test) (Sokal and Rohlf 1981) and variance homogeneity (Levene's test) (Levene 1960). For this, we compared the abundance of boring sponges per coral reef in 2005/2006 versus the same reefs in 2009/2010. Differences in substrate invasion were analyzed using a one-way analysis of variance (ANOVA). If the results of the ANOVA revealed a significant difference, a post-hoc analysis (Newman–Keuls test) was then performed to evaluate the differences observed. The level of significance was 5% (*P* < 0.05).

## Results

### Bleaching and water temperature

**Figure 3:**
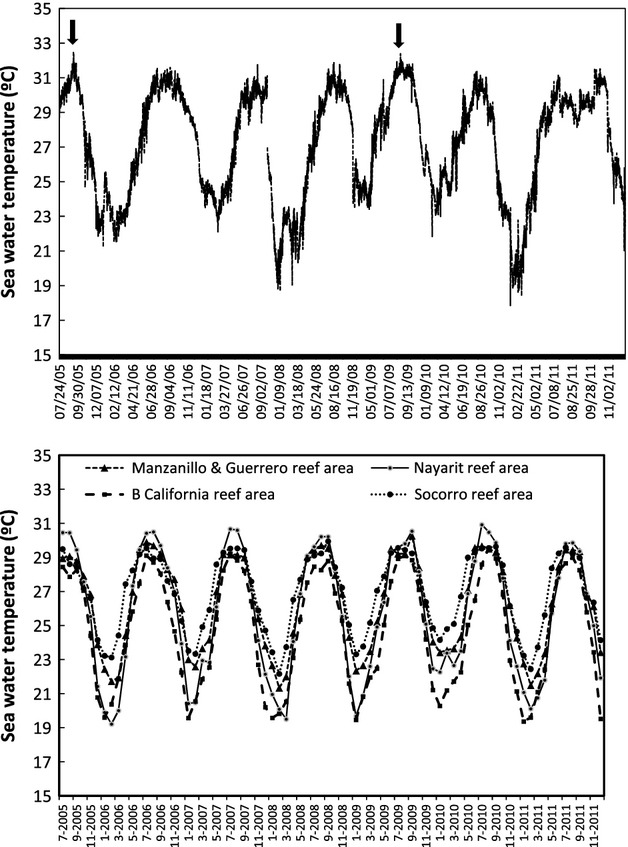
Above. Average daily seawater temperature from June 2005 to November 2011 at 5 m depth on Bahía Tiburones reef (Isabel Island). Gaps in the data represent failure of the data loggers. Arrows show positive anomalies that induced bleaching events (see results).Below. Sea surface temperature (SST) for the different reef areas studied

Water temperature during the study showed an evident annual seasonal pattern, with high values in spring/summer and low values in autumn/winter (Fig. 3, above). However, two positive anomalies were detected in summer 2005 and 2009 at Isabel island. In the former, an increase in 1°C for 4 weeks induced partial bleaching, but not coral mortality (see Fig. 2), but during summer of 2009, an increase in almost 1.5°C for 6 weeks above the average for the season led to a severe coral bleaching in the corals from Isabel Island (100% bleached coral cover) (Fig. 2A), and partial bleaching (only the tips of the branches in Punta Mita), but apparently there was no mortality (see Discussion).

### Boring sponges abundance and habitat

**Figure 4:**
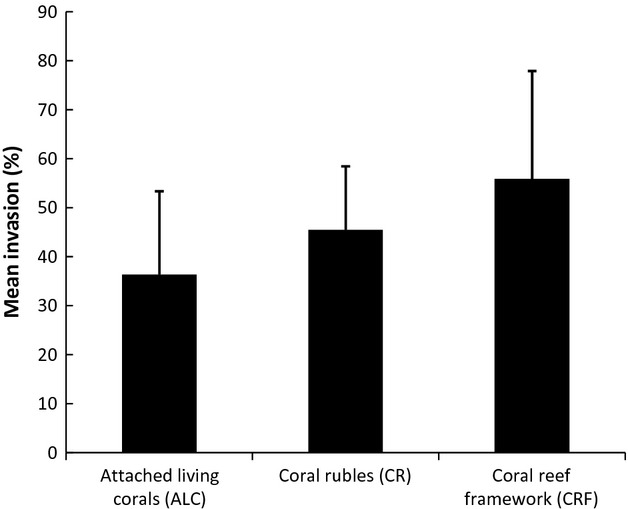
Comparison of the mean boring sponge invasion (± SE) in the different coral reefs substrata counting the total of the samples analyzed (see Table 2).

We found a high percentage of invasion in all coral reefs, as 45.8% of the more than 10000 coral samples examined were invaded by boring sponges. In general, reefs that were previously impacted by bleaching events had the highest values (Table 1), and the most invaded substrata was the coral reef framework (56%) (Fig. 4, Table 2).

**Table 2:** Summary of the one-way ANOVA for differences in boring sponge invasion in attached live coral (ALC), coral reef framework (CRF), and coral rubble (CR) (see Fig. 4)

Source	Sum of Squares	Df	Mean Square	F-Ratio	*P*-Value
Between groups	3550,7	2	1775,35	4,31	**0,0183**
Within groups	22656,4	55	411,08		
Total (Corr.)	26207,1	57			

Student Newman Keuls Tests					

Contrast	Significant difference		Difference		± Limits
CR versus CRF			−10,4613		13,6428
CR versus ALC			9,09045		12,5668
CRF versus ALC	*		19,5517		13,3643

denotes a statistically significant difference.

**Figure 5:**
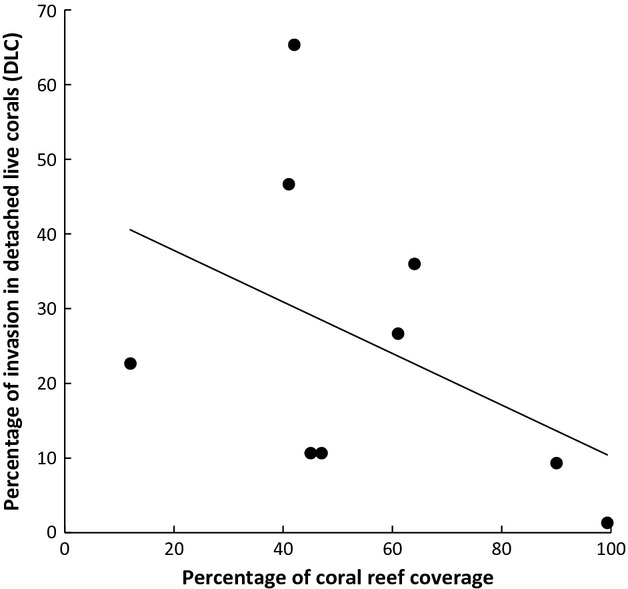
Relationship between the percentage of invasion by boring sponges in colonies recently detached from the matrix (*y*-axis) and live coverage of the reef (*x*-axis). Although there is not a significant correlation, the line representing the ‘best fit’ for the data shows that in well preserved coral reefs with a high coral coverage (to the right of the plot) the invasion by sponges in the detached colonies are lesser than in bad preserved reefs (to the left). Please note that this information is only available in some reefs because we did not always find colonies recently broken away from the matrix to make this analysis.

It is also remarkable that most of the detached live colonies had their stem invaded by sponges, and these colonies were mainly found in reefs with evident signs of deterioration, such as ample areas of the original coral framework largely fragmented and low cover of live colonies (Fig. 2C, Fig. 5).

**Figure 6:**
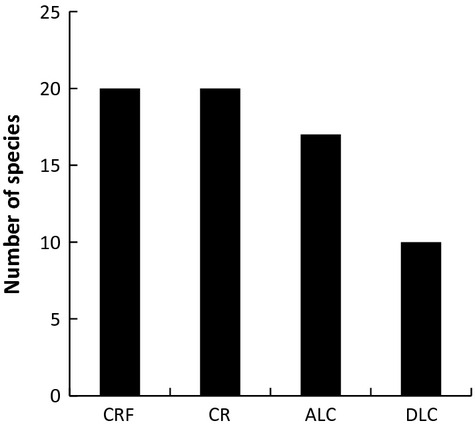
Total number of species found in each substrata (CRF: Coral Reef Framework, CR: Coral rubbles, ALC: Attached Live Corals, DLC: Detached Live Coral).

With 20 species described so far, boring sponges can be considered very diverse in Mexican coral reefs. The coral framework together with coral rubbles harbored the highest species richness, with 20 species each, and the coral colonies (attached and detached) had the lowest richness; 17 and 10 spp, respectively (Fig. 6). However, it seems that none of these species has the capacity to bore through the coral tissue. Species richness also varied greatly between reefs. Reefs that have lost most of their framework, such as Antiguo Corral del Risco, Cabo Pulmo, Bahía Tiburones, San Gabriel, and Guayabitos, or reefs completely devastated, such as Careyeros, currently made up of only fragments of the original reef framework, presented the highest number of species (from 8 to 13 species). In contrast, better preserved coral reefs, such as the coral reefs from Oaxaca (La Entrega, San Agustin) and Guerrero (Ixtapa Island and Playa Manzanillo), had the lowest number of species (from 5 to 7 species).

**Figure 7:**
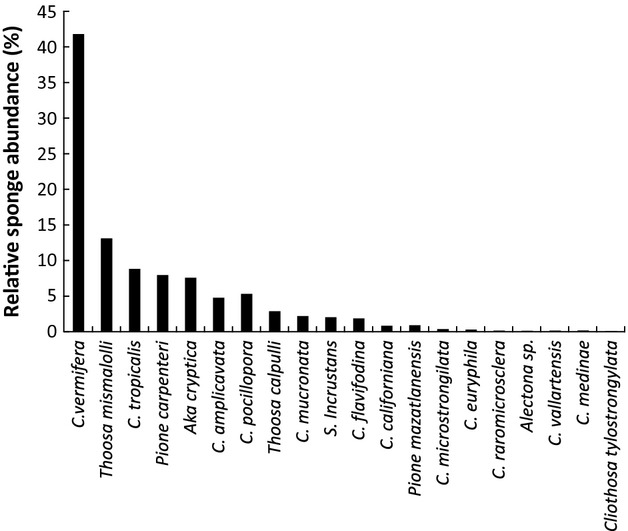
Relative species abundance expressed as a percentage across all reefs and years, arranged from most (*Cliona vermifera*) to least abundant (*C. tylostrongylata*)

Regarding the abundance, the most important species was *Cliona vermifera*, which was found in 42% of the samples analyzed, followed by *Thoosa mismalolli*, present in 13% of the fragments, *Cliona tropicalis* in 9%, and *Pione carpenteri* in 8% (Fig. 7). There was also a group of species such as *C. californiana, C. euryphylla*, *C. raromicrosclera*, *C. vallartense*, *C. medinae* and *Cliothosa tylostrongylata* that could be considered accidental due to their low abundance in these ecosystems. In the detached colonies, the most abundant species was *C. vermifera* (41.4%), followed by *C. tropicalis* (17.6%), *A. cryptica* (15.6%), and *P. carpenteri* (11.3%).

**Figure 8:**
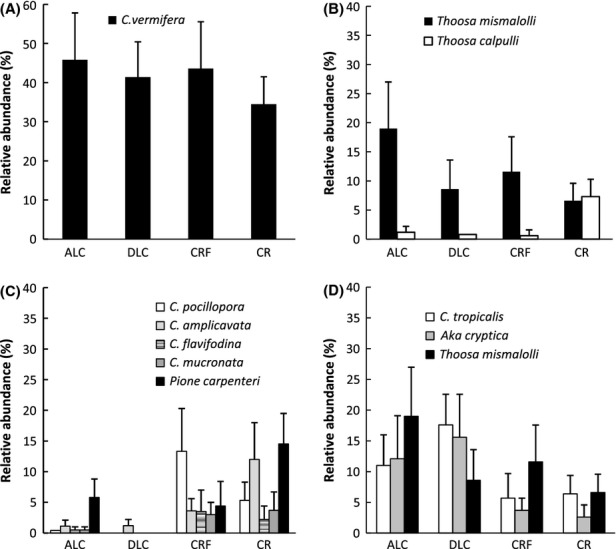
Relative species abundance of some boring sponges in the different substrata. (A) *Cliona vermifera*, a species that does not show any preference for the substrata. (B) *Thoosa mismalolli* and *T. calpulli*, two species of the same genera with different substrata affinity. (C) Group of species that prefer to live in dead coral. (D) Group of species that prefers live in living coral (ALC**:** Attached Live Corals, DLC**:** Detached Live Coral, CRF**:** Coral Reef Framework, CR**:** Coral rubbles). Please note the different scale in the case of *C. vermifera*.

*Cliona vermifera* is the most abundant and amply distributed species. As shown in Fig. 8A, *C. vermifera* does not show any preference of habitat, and it can live in the same way in coral colonies, unstable coral rubble, and coral framework. In contrast, there are others species, such as *C. tropicalis* that prefer to live in the basal stem of the coral colonies instead of in the coral rubble (Fig. 8D). There is an important group of species (*Pione carpenteri*, *C. pocillopora*, *C. flavifodina*, or *Thoosa calpulli*) that prefer to live inside of coral rubble or in the matrix (Fig. 8C). Here, it is also remarkable that the two species of the genus *Thoosa* use different habitats; *T. mismalolli* lives mainly inside live colonies and *T. calpulli* prefers rubbles (Fig. 8B).

### Temporal variation

**Figure 9:**
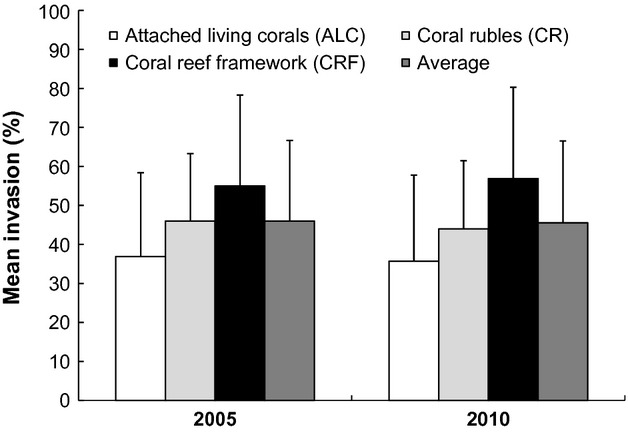
Comparison of the mean boring sponge invasion (± SE) in different coral reefs substrata between 2005 and 2010. Bars represent the mean bioerosion for each substrata.

The results suggest that invasion has not increased significantly in coral reefs in the last few years in any of the habitats studied (substrata) (Fig. 9, Table 3). There is no clear pattern in the temporal variation in the main species either; only the abundance of *C. vermifera* and *Aka cryptica* seemed to have increased over time in some reefs, especially in those that suffered the impact of El Niño events.

**Table 3:** Summary of the one-way ANOVAs for temporal differences (2005 vs 2010) in boring sponge invasion in coral reefs (live coral colony, coral reef framework, and coral rubble) (see Fig. 9)

Source	Sum of Squares	Df	Mean Square	*F*-Ratio	*P*-Value
Live coral colony (LCC)					
Between groups	828,409	1	828,409	0,02	**0,8962**
Within groups	9486,62	20	474,331		
Total (Corr.)	9494,91	21			
Coral reef framework (CRF)					
Between groups	142506	1	142506	0,03	**0,8762**
Within groups	7926,86	14	566204		
Total (Corr.)	7941,11	15			
Coral rubbles (CR)					
Between groups	161,333	1	161,333	0,05	**0,8198**
Within groups	5432,24	18	301,791		
Total (Corr.)	5448,38	19			

## Discussion

### Relationship of bioerosion by sponges and bleaching: a global perspective

Boring sponges account for 70% or more of bioerosional damage to skeletons of hermatypic corals in both the Western Pacific and Caribbean regions, accounting for 65–90% of total bioerosion occurring within the first 2 cm of coral skeleton (Highsmist 1981). We do not have similar information in the east Pacific coral reefs yet, but the fact that 46% of the fragments of the coral studied were invaded by boring sponges suggests that they are currently playing a significant role in the destruction of the physical structure of Mexican Pacific corals, which could extrapolate to the eastern Pacific Ocean, since the study area represents 46% of the coral distribution of the Eastern Pacific Ocean.

**Figure 10:**
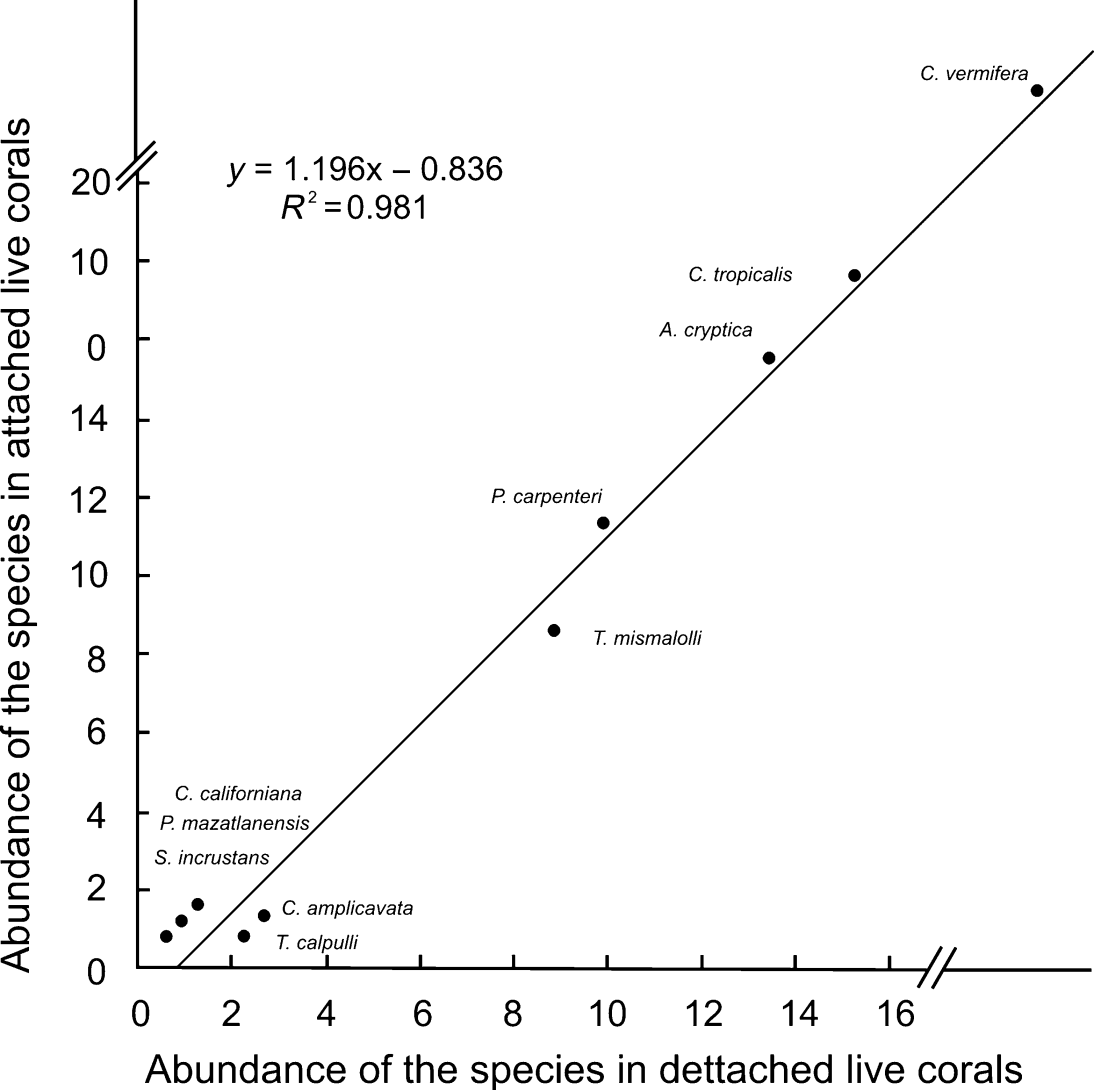
Relationship between the abundance of the main boring sponges in attached and in recently detached colonies. Please note the broken Y-X axis to include the species *Cliona vermifera* (Y coordinate: 41.4; X- coordinate: 39.3), which is out of the range due to its high abundance. Lineal model and coefficient of determination *R*^2^ are included inside the plot (*P* < 0.01).

**Figure 11:**
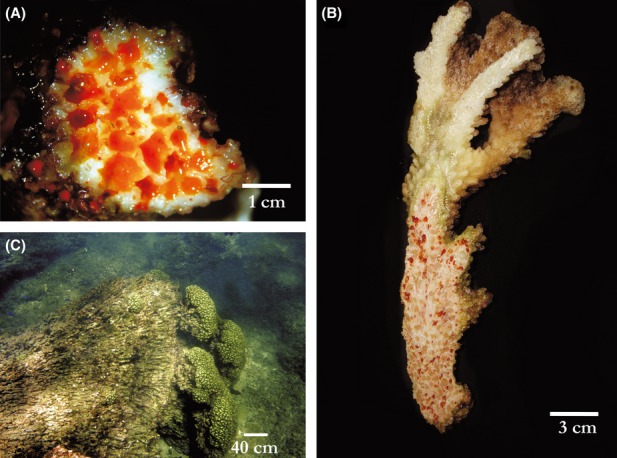
(A) Cross-section, and (B) Longitudinal section of a coral branch showing the chambers formed by *Cliona vermifera*) (C) Large portion of the coral framework heavily invaded by boring sponges, dislodged after a storm.

Thus, the most important results of this study was the high diversity (20 species) and abundance of boring sponges (46% of the fragments were invaded) that we found in all coral reefs, especially in those that suffered the massive bleaching during El Niño 97/98, such as Careyeros, Antiguo Corral del Risco, and Cabo Pulmo (Table 1). It is known that bleaching episodes can cause catastrophic loss of coral cover, thus increasing the available habitat and creating new one for sponge colonization (Sammarco 1980, 1982). This kind of the sponges prefer the dead portions of the reef framework and the exposed basal region of the colonies to avoid the chemical defense of corals, and to increase the chance of sponge invasion by larvae or fragments (Pari et al. 2002; López-Victoria and Zea 2005; Nava and Carballo 2013), which is probably the cause of such high richness in Eastern Pacific coral reefs. In a next step, after invading the coral, the sponge excavates an extensive system of cavities and tunnels, which can weaken the reef by causing dislodgment of the reef framework, especially during wear and tear caused by waves. The strong relationship between the composition and abundance of boring sponges in attached and in recently detached colonies (Fig. 10) indicates that these colonies were invaded before they were broken away from the coral framework. Thus, these results clearly suggest that the boring sponges are weakening the union between the colony and the reef framework (see Fig. 11), and this dislodgment of live colonies is probably more pronounced in deteriorated coral reef.

On the other hand, although sponge abundance has not increased over time, the high levels of sponge invasion recorded in almost all the reefs studied seem to support our hypothesis. Massive bleaching have been recorded in Mexican pacific coral reefs several years before our study, the main one after El Niño 1997/98 (Carriquiry et al. 2001), which caused major reductions in the coverage in some areas from the south (Bahías de Huatulco), center (Bahía Banderas) and north Pacific (Baja California), where cover of important reef, such as Antiguo Corral del Risco or Careyeros, were completely lost. Even reefs, such as Cabo Pulmo, despite being declared a Protected Marine Area, lost much of its pre-1997 coverage (from 62% to 18%) (Table 1). Later, others unrecorded local bleaching episodes, even associated with cold waters have occurred in some of these reefs after 1997 (Cupul-Magaña and Calderón-Aguilera 2008; Hernández et al. 2010); which have brought about the gradual loss of coral cover and the subsequent increasing in boring sponges until the high values recorded currently.

There are also different examples worldwide that support our hypothesis. For example, the abundance of boring sponges significantly increased (up to 150%) after an important loss of coral cover by strong bleaching events in the central Great Barrier Reef (Schönberg and Ortiz 2008). Something similar occurred after the El Niño 1997/98 event, which reduced extensive areas from the Arabian Gulf, South Africa, and the Cayman Islands, and produced an increase in boring sponges (Riegl 2001), which rose up to 81% due to the availability of dead coral in Maldives, Seychelles, and Chagos (Sheppard et al. 2002). This was also the main cause of the high richness and abundance of boring sponges in the atolls from Lakshadweep (India), where mortality of corals due to sponge attack was as high as 80% (Thomas 1989). In the Caribbean, drivers of both coral mortality and erosion have operated as virtually chronic pressures throughout the entire region in recent decades (see Alvarez-Filip et al. 2011), which have reduced coral cover from 50% to 10% (Gardner et al. 2003), and although we do not have much information about invasion by boring sponges in this region, we know that the sponge *Cliona langae* overgrew 10.8% of the total area of the populations of the elkhorn coral of La Parguera (Puerto Rico) after the bleaching of 1987 (both dead and living corals) (Williams et al. 1999).

### Boring sponges as bioindicators of coral reefs health?

Indicator species are species indicative of the status of a larger functional group of species, and can be used to assess the condition of the environment, to provide early warning signals of changes in the environment, or to diagnose the cause of an environmental problem (Beyeler and Dale 2001). The proliferation of particular species -like the case of the La Parguera- after an impact seems to be a recurrent fact in other reefs. Thus, when corals are stressed, some of these species, normally the most abundant in the area, gain advantage of the new situation and proliferate. The same occurred with *Cliona caribbea*, which increased in abundance in the Caribbean coast of Costa Rica after the 1983 coral-bleaching event (Cortés et al. 1984). The abundance of this species has also increased in the last decades in Belize coral reefs in relation to water warming and catastrophic events, such as hurricanes, which were also responsible for the decrease in live coral over the past 20 years (Cortés et al. 1984). This seems to be the situation of *Cliona vermifera*, which have increased in some Mexican coral reefs affected by beaching phenomena (Chávez-Castro 2011). In fact, this is the most common species more than three times as abundant as the next species *Thoosa mismalolli* (Fig. 7). *C. vermifera* is also one of the most abundant bioeroding sponges in Costa Rica (Guzmán 1986), which suggests that this species is a dominant boring sponge from the Eastern Pacific coral reefs. The niche differentiation has been strongly evident in boring sponges (Hartman 1957), and the high abundance of *C. vermifera* could be explained in part by a wide niche, evident in the lack of preference by a specific type of coral substrata (Fig. 8A). These two species together with *C. tropicalis* and *Aka cryptica* dominate the total bioeroding assemblages in Mexican Pacific coral reefs. Something similar occurs in the Caribbean, where four species; *C. aprica*, *C. caribbea*, *C. delitrix* and *C. tenuis* dominate up to 90% of the bioeroding assemblages (Rose and Risk 1985; Rützler 2002; MacDonald and Perry 2003; López-Victoria et al. 2006).

Selection of an effective indicator is key to the overall success of any monitoring program in coral reefs, and boring sponges is an important functional group that should be included in any coral reefs monitoring program because they reflect stress-caused changes in ecosystem structure (Carballo et al. 1994).

### Climate change, boring sponges, and coral reefs

Coral reefs are vulnerable ecosystems that have shown great sensitivity to the unusually warm water temperatures associated with climate change (Hoegh-Guldberg et al. 2007; Baker et al. 2008). Many reefs from the East Pacific, including the Mexican one, have lost their frame-building corals during past ENSO events (Glynn 1988, 1990, 2000; Glynn and Ault 2000), and recently, a new kind of ENSO, called Modoki, which unlike its eastern relative is centered in the central pacific seems to be associated with global warming (Ashok and Yamagata 2009). One possible side effect of the rising of the global temperatures associated with global warming might be more frequent and intense El Niño events, which may suppress the ability of corals to recover at all from mass bleaching.

Although boring sponges do not always thrive in conditions that are adverse for coral (Nava and Carballo 2013), the stressful conditions associated with bleaching episode favors to sponges, which unlike corals, appear to continue to function normally or can even occasionally become epidemic (Rose and Risk 1985; Vicente 1990). It has been reported that sponges are more bleaching-resistant than corals (Vicente 1990), and it has been demonstrated that many successful and aggressive species of bioeroding sponges, such as *C*. cf. *orientalis*, harbor symbiotic dinoflagellates, which displayed a more stable photochemical efficiency during and after heat stress than corals, such as *Acropora palifera* (Schönberg and Loh 2005). *C*. cf. *orientalis* is one of the most abundant species in the Indo Pacific Ocean, and this characteristic gives it a higher survival potential than corals under heat stress like that caused during ENSO events (Schönberg et al. 2008). In fact, *C*.cf. *orientalis*is is a very aggressive species whose massive forms cover several meters in diameter (Schönberg 2001). In consequence, the temperature increase in sea surface, that can be harmful for corals, may indirectly increase the expansion of some of these sponges (Rützler 2002; Márquez et al. 2006).

On the other hand, acidification is also increasing in the world's oceans (Hoegh-Guldberg et al. 2007), which could have major implications for the future of the eastern tropical Pacific reef, which are poorly cemented and prone to intense bioerosion (Manzello et al. 2008).

Sponges are able to dissolve part of the carbonate during the bioerosion process (Rützler and Rieger 1973), for that the sponge lowers the pH at the tissue-substrate interface where specialized etching cells act (chemical boring) (Pomponi 1977). *Pione* cf. *vastifica* dissolves three masses of reef CaCO_3_ framework per each part of carbonate removed mechanically (Zundelevich et al. 2007), and this proportion is also high in *C. vermifera* and *C. flavifodina* (27% and 10%, respectively) (Nava and Carballo 2008). As this process is conducted extracellulary, the lower the environmental pH is to begin with, the less pronounced is the gradient between ambient sea water and the site of dissolution, and the lower will be the metabolic cost required for bioerosion. Recently have been proved that the chemical erosion increase significantly under more acidic conditions. This finding implies that tropical reef ecosystems are facing the combined effects of weakened coral calcification and accelerated bioerosion (Wisshak et al. 2012).

Modern scleractinian corals have existed on earth for over 240 million years (Stanley 2003), and currently many of them are not constructing more reefs and therefore their reef framework is being lost due to bioerosion. Cool, CO_2_-rich upwelling water masses at eastern Pacific area lead to naturally low pH and saturation states, that together with the associated decrease in coral calcification rate, as a result of lower aragonite saturation state, is likely to reduce the ability of reef builders to prevent and/or repair damage caused by bioeroders (Langdon et al. 2000). Consequently, the effects of coral bleaching and mortality on bioerosional rates and processes are likely to be magnified as a result of interacting climate change stressors, which further decrease reef framework strength and depress reef accumulation rates. If the present trends of environmental deterioration continue, it is logical to expect a rise in sponge bioerosion promoted by the increase in dead coral framework (Schönberg and Ortiz 2008).
